# Biofabrication of nanocomposite-based scaffolds containing human bone extracellular matrix for the differentiation of skeletal stem and progenitor cells

**DOI:** 10.1007/s42242-023-00265-z

**Published:** 2024-03-05

**Authors:** Yang-Hee Kim, Janos M. Kanczler, Stuart Lanham, Andrew Rawlings, Marta Roldo, Gianluca Tozzi, Jonathan I. Dawson, Gianluca Cidonio, Richard O. C. Oreffo

**Affiliations:** 1 Faculty of Medicine, Bone and Joint Research Group, Centre for Human Development, Stem Cells and Regeneration, Institute of Developmental Sciences, University of Southampton, Southampton, SO16 6YD UK; 2 School of Pharmacy and Biomedical Science, University of Portsmouth, Portsmouth, PO1 2DT UK; 3 School of Engineering, Faculty of Engineering and Science, University of Greenwich, Greenwich, ME4 4TB UK; 4 Center for Life Nano- and Neuro-Science (CLN2S), Italian Institute of Technology, 00161 Rome, Italy

**Keywords:** Extracellular matrix, Nanoclay, Bone, 3D bioprinting

## Abstract

Autograft or metal implants are routinely used in skeletal repair. However, they fail to provide long-term clinical resolution, necessitating a functional biomimetic tissue engineering alternative. The use of native human bone tissue for synthesizing a biomimetic material ink for three-dimensional (3D) bioprinting of skeletal tissue is an attractive strategy for tissue regeneration. Thus, human bone extracellular matrix (bone-ECM) offers an exciting potential for the development of an appropriate microenvironment for human bone marrow stromal cells (HBMSCs) to proliferate and differentiate along the osteogenic lineage. In this study, we engineered a novel material ink (LAB) by blending human bone-ECM (B) with nanoclay (L, Laponite^®^) and alginate (A) polymers using extrusion-based deposition. The inclusion of the nanofiller and polymeric material increased the rheology, printability, and drug retention properties and, critically, the preservation of HBMSCs viability upon printing. The composite of human bone-ECM-based 3D constructs containing vascular endothelial growth factor (VEGF) enhanced vascularization after implantation in an ex vivo chick chorioallantoic membrane (CAM) model. The inclusion of bone morphogenetic protein-2 (BMP-2) with the HBMSCs further enhanced vascularization and mineralization after only seven days. This study demonstrates the synergistic combination of nanoclay with biomimetic materials (alginate and bone-ECM) to support the formation of osteogenic tissue both in vitro and ex vivo and offers a promising novel 3D bioprinting approach to personalized skeletal tissue repair.

**Graphic abstract:**

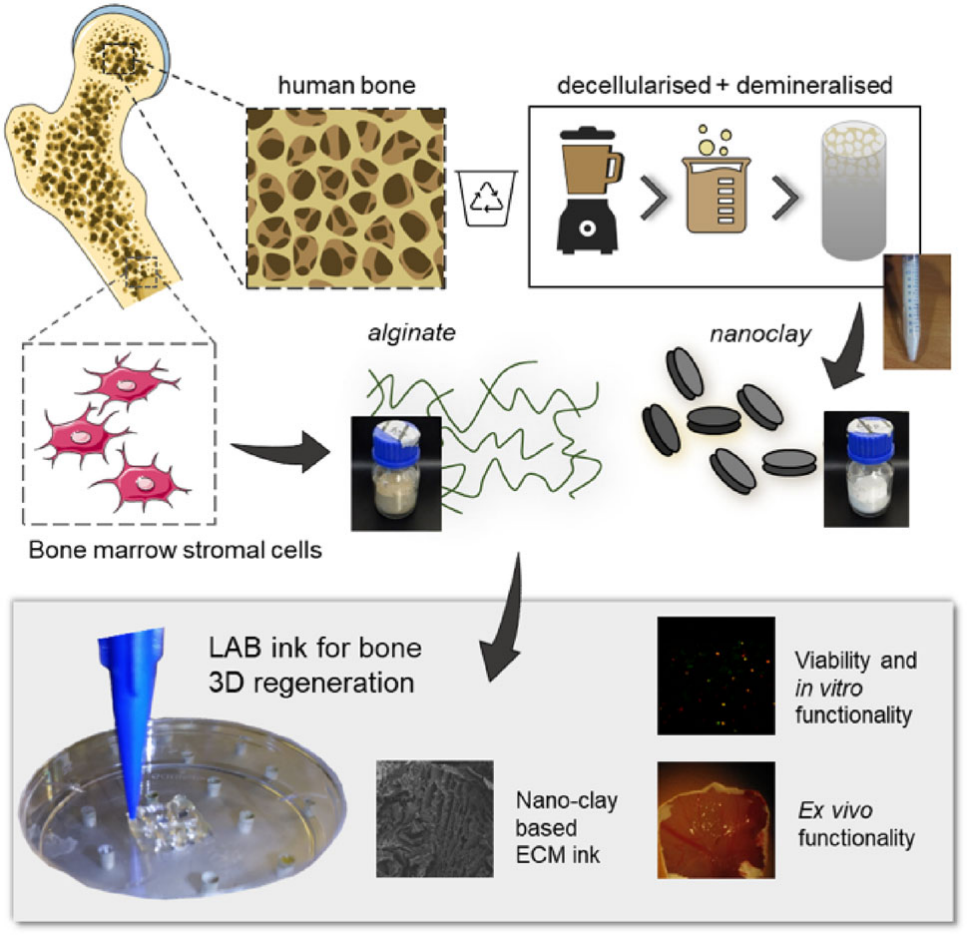

**Supplementary Information:**

The online version contains supplementary material available at 10.1007/s42242-023-00265-z.

## Introduction

Skeletal tissue engineering (TE) provides functional tools for repairing damaged or diseased bone tissue. Over the last decade, biofabrication approaches for TE have explored a number of biomaterials that support cell delivery and sustain the release of biological agents of interest for the repair [1–4] or the modeling [5] of bone.

However, to date, material inks formulated using either natural or synthetic platforms have been unsuccessful in fully supporting skeletal repair and resembling/recapitulating the native bone microenvironment [6, 7]. Recently, organic nanofillers have shown significant promise in enhancing printability and skeletal functionality [3]. Particularly, nanoclays have been employed to engineer a library of material inks capable of sustaining skeletal stem and progenitor cell viability and differentiation in vitro [8], ex vivo [9, 10], and in vivo [11]. These nanoclay composites provide a powerful tool for engineering a rapidly evolving skeletal microenvironment. However, nanocomposite materials alone cannot fully recapitulate or mimic the native skeletal microenvironment, limiting the biomimetic platform for stem-progenitor cell differentiation and skeletal maturation.

The physicochemical composition of the native bone tissue is ideal for skeletal repair.

Indeed, bone extracellular matrix (bone-ECM) contains several growth factors (GFs) (e.g., bone morphogenetic protein-2 (BMP-2) and others) and polymeric constituents (e.g., collagen), essential for the development and repair of skeletal tissue [12–14]. Autologous and allogenic bone grafts are routinely used clinically to repair large skeletal defects. Impaction bone grafts are used to repair segmental defects by harnessing cadaveric tissue. Nevertheless, (i) the scarcity of available bone tissue, (ii) the lack of donor-to-donor compatibility, and (iii) the functional ability to match the defective architecture and regenerative capacity have limited the use of these human-derived bone grafts. Moreover, the inability of impaction bone grafts to fully facilitate bone regeneration remains a limitation.

A potential solution to these issues is the application of biomaterials engineered from native skeletal tissues. Using decellularized allografts, native ECM material can be isolated together with the removal of any allogeneic cellular components and epitopes that could trigger an immune response upon implantation. Recent decellularization techniques have facilitated the preparation of ECM derived from tissue previously difficult to digest and process. However, human-based decellularized ECM tissues have not yet been successfully applied in skeletal TE applications. Xenogenic ECM materials have been explored as printable inks to support tissue-specific repair, harnessing the physiological mechanisms from naturally derived matrices [15]. A number of studies in the last decade [16] have attempted to isolate ECM-based materials from animal tissues, including cardiac [17] and liver [18] tissues. Nevertheless, human applications of animal-derived ECM material inks are limited due to species differences and immunologic considerations. Currently, the immunogenicity of animal-derived materials limits their clinical translation due to the natural immune reaction observed upon implantation. Human-based materials have tremendous clinical potential given their allogeneic nature and innate biocompatibility [19]. Moreover, human-derived matrices can be used to encapsulate, differentiate, and guide the fate of stem cells, but these properties remain poorly explored. Currently, tissue-derived ECM materials have failed to function effectively as a reproducible bio-printable platform due to: (i) complex matrix derivation steps, typically involving acidic components and extensive filtering procedures, (ii) poor viscoelastic properties of the derived materials, limiting extrusion-based bioprinting approaches, and (iii) species-level differences in the ECM composition of animal and human sources, which cause host-immune response issues upon implantation [20, 21]. Thus, a human-sourced ECM material ink could potentially shift the paradigm in bioink design by offering an innovative approach to personalized skeletal regenerative medicine [22].

**Fig. 1:**
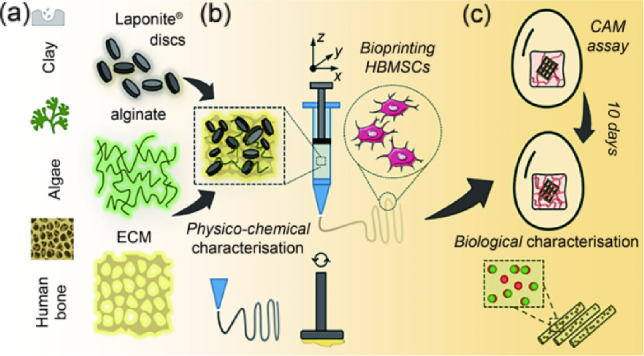
A novel biomaterial ink system engineered from the combination of nanoclay disks, alginate and a novel demineralized and decellularized ECM from human bone (**a**). The nanocomposite ink rheological properties (**b**) were investigated, along with the ability of the nanocomposite ink to be printed with increased resolution in three dimensions. The inclusion of HBMSCs allowed analysis of viability and differentiation over 21 days, as well as evaluation and demonstration of 3D functionality in a CAM model (**c**). ECM: extracellular matrix; HBMSCs: human bone marrow stromal cells; 3D: three-dimensional; CAM: chick chorioallantoic membrane

The current study demonstrates the printing capacity, in vitro stability, and ex vivo functionality of a novel human bone ECM-based bioink composite. The inclusion of a nanoclay filler was found to improve the physicochemical properties limiting the swelling rate and porosity while enhancing the material viscosity profile (Fig. 1a). Three-dimensional (3D) printing of the human bone marrow stromal cell (HBMSC)-laden bone-ECM material resulted in a stable culture that supported cell growth and promoted skeletal cell functionality in vitro (Fig. 1b) and ex vivo (Fig. 1c). The inclusion of nanoclay particles was supportive for ex vivo drug retention compared to the clay-free controls, providing a platform able to support vascular and bone regeneration. This biomimetic nanocomposite material offers a promising 3D bioprinting approach for personalized skeletal tissue repair.

## Materials and methods

### Nanocomposite hydrogels preparation

Nanocomposite hydrogels were prepared in a sterile class II cell culture hood. Laponite^®^ (LAP, L, XLG grade, BYK Additives & Instruments, UK) was allowed to disperse at either 3% or 4% (30 or 40 mg/mL respectively) in deionized water (DW) for 3 h under constant stirring until clear, followed by ultraviolet (UV) sterilization. The bone-ECM was prepared using a previous protocol [14]. Briefly, we collected cancellous bone fragments from donated femoral heads from patients undergoing total hip-replacement for osteoarthritis with full national ethical approval following informed patient consent (Southampton General Hospital, University of Southampton under approval of the Southampton and Southwest Hampshire Research Ethics Committee (Ref No. 194/99/1)), using a bone nipper and washed with 2% penicillin/streptomycin (P/S). Bone fragments were ground to a fine powder and stirred in 0.5 N HCl at room temperature for 24 h to allow complete demineralization, as previously reported [23]. The demineralized bone matrix (DBM) was fractionated using a 45 µm-pore sieve and washed with DW. A mixture of chloroform and methanol (1:1) was used to treat the DBM for 1 h to extract the lipidic portion. The lipid-free DBM was subsequently lyophilized overnight and stored at −20 °C for future use. To deplete the cellular component of the DBM, a 0.05% Trypsin and 0.02% ethylenediaminetetraacetic acid (EDTA) solution was added to the DBM and stirred at 37 °C in a 5% CO_2_ incubator for 24 h. The decellularized DBM was further rinsed and treated in pepsin solution (20 mg ECM/1 mL of pepsin solution) under constant agitation at room temperature for seven days, followed by centrifugation. The supernatant (referred to as decellularized matrix–ECM (B)) was collected and lyophilized. Full characterization of the human decellularized bone extracellular matrix was provided by Kim et al. [14].

The lyophilized bone-ECM (B) was added at a concentration of 10 mg/mL to a Laponite^®^ (L) suspension. Following 2 h stirring at room temperature, alginate (A, alginic acid sodium salt from brown algae, Sigma, UK) was added to the Laponite-bone-ECM (LB) suspension and homogenized with a spatula for 8–10 min to allow alginate inclusion. The combinations of LAP, alginate and bone-ECM examined in this study are detailed in Table 1. Laponite-alginate-bone-ECM (LAB) ink was stored at room temperature and printed the following day.

**Table 1:** Schematic of the composite ink combinations used in this study

Polymer	Content					
L: Laponite^®^ (g/mL)	0.03			0.04		
A: alginate (g/mL)	0.06	0.08	0.10	0.06	0.08	0.10
B: human bone-ECM	10 mg/mL					

Laponite^®^ (L) and alginate (A) were mixed with human bone-ECM (B) to generate material composites for further physicochemical characterization; ECM: extracellular matrix

#### Physicochemical characterization

To investigate the effect of Laponite^®^ (L) on alginate (A) and bone-ECM (B), the mass loss and swelling ratio of the nanocomposite gels were investigated as shown previously [24].

LAB hydrogels with various concentrations of L and A (Table 1) were prepared and cast in 500 µL molds. To obtain the initial wet mass, the samples (*n*=3) were weighed before (${m}_{{\text{initial}}}$) and after crosslinking (${m}_{{\text{initial}}, {\text{t}}0}$). LAB samples (*n*=3) were lyophilized to obtain their dry weights (${m}_{{\text{dry}},\mathrm{ t}0}$). The macromer fraction was calculated as follows:

1 $$\mathrm{macromer\,\, fraction}=\frac{{m}_{{\text{dry}},\mathrm{ t}0}}{{m}_{{\text{initial}},\mathrm{ t}0}}.$$

The remaining samples (*n*=3) were incubated at 37 °C in phosphate-buffered saline (PBS, Thermo-Fisher, UK) or Hank’s balanced salt solution (HBSS, Thermo-Fisher). The samples were reweighed (${m}_{{\text{swollen}}}$) after 24 h. LAB samples were subsequently lyophilized and weighed (${m}_{{\text{dry}}}$). The sol fraction was calculated using Eqs. (2) and (3). The mass swelling ratio (*q*) was calculated using Eq. (4).

2 $${m}_{{\text{initial}},\mathrm{ dry}}= {m}_{{\text{initial}}}(\mathrm{actual\,\, macromer\,\, fraction}),$$

3 $$\mathrm{sol fraction}=\frac{{m}_{{\text{initial}},\mathrm{ dry}}-{m}_{{\text{dry}}}}{{m}_{{\text{initial}},\mathrm{ dry}}}\times 100\mathrm{\%},$$

4 $$q= \frac{{m}_{{\text{swollen}}}}{{m}_{{\text{dry}}}}.$$

#### Scanning electron microscopy

Scanning electron microscopy (SEM, FEI Quanta 200 FEG) at a voltage of 5 kV (spot size 3) was used to image the acellular gels. Samples were dehydrated using a freeze-drier (Lablyo Mini, Froze in Time Ltd., UK) for 12 h and platinum-coated to allow SEM analysis (Q150TES, sputter coater, UK). Porosity was calculated from SEM images (*n*=3) using the ImageJ software [25].

#### Rheological measurements of nanocomposite hydrogel properties

The rheological properties of the nanocomposite hydrogels were carried out using a cone-plate rheometer (MCR92, Anton Parr, UK) at room temperature with a 0.1 mm gap. The viscosity (Pa·s) of the LAB ink formulations and controls was measured using shear rates ranging between 1 and 100 s^−1^ with a linear increase. The stable viscosity of the inks was measured applying a constant shear rate (10 s^−1^) for 720 s. Considering a viscoelastic behavior at 1% shear strain, frequency sweeps were performed over a range of 0.01–100 s^−1^. Storage and loss moduli of controls and LAB material inks were acquired at 1% shear strain.

#### Printing fidelity

The fidelity of filament deposition was assessed as previously published [26]. Briefly, three layers were deposited, resulting in layering strands at an increasingly larger distance. Immediately after deposition, the images of the scaffolds (*n*=3) were captured using a light stereomicroscope (Zeiss, Germany) equipped with a Canon Powershot G2 camera and analyzed using ImageJ to identify the fused segment length (fs), filament thickness (ft), and filament distance (fd). The results were plotted as the ratio of fs and ft as a function of fd*.*

#### Printing of the nanocomposite ink

LAB inks were deposited to investigate the printing fidelity, as shown previously [26]. Briefly, the LAB inks were printed in a winding pattern with exponentially increasing strand distances and imaged (Stemi DV4, Zeiss, UK) immediately after printing. Images were analyzed with ImageJ software to obtain the actual strand distance, fused segment length, and strand width. The filament fusion test was then plotted based on the quotient of segment length and strand width as a function of strand distance. An in-house bioprinter [10] was used to deposit acellular and cell-laden LAB inks using a 410 µm nozzle (Fisnar Europe, UK). Multi-layer scaffolds (10 mm×10 mm) were printed in an alternating pattern (ABAB, 0°/90°) with a layer height of 350 μm and a strand distance of 2 mm. The printed structures were crosslinked for 10 min using 100 mmol/L CaCl_2_ solution. Scaffolds for viability (totally *n*=12) and functionality (totally *n*=16) tests were printed with *n*=3 scaffolds used at each time point.

#### Cell isolation, encapsulation, and printing

Unselected HBMSCs were isolated as previously described [12] from patients undergoing total hip replacement with full national ethical approval following informed patient consent (Southampton General Hospital, University of Southampton under approval of the Southampton and Southwest Hampshire Research Ethics Committee (Ref No. 194/99/1)). Briefly, to remove excessive fat, the bone marrow aspirate was resuspended and washed in alpha-modified Eagle’s medium (α-MEM), filtered through a 40-µm cell strainer, and layered on LymphoPrep™ (Lonza) using density centrifugation at 2200 r/min (800 g) for 40 min at 18 °C. The portion of bone marrow mononuclear cells (BMMNCs) was isolated and plated in cell culture flasks and maintained at 37 °C and 5% CO_2_ balanced air with α-MEM supplemented with 10% (volume fraction) fetal bovine serum (FBS), 100 U/mL penicillin and 100 µg/mL streptomycin (Pen/Strep). Cells were passaged at approximately 80% cell confluency using collagenase IV (200 mg/mL) in serum-free media and then treated with trypsin-ethylenediamine tetra-acetic acid. HBMSCs were used for experimental studies at passage two. To visualize the cells after printing for viability studies, cells were suspended at a density of 1×10^6^ cells /mL in serum-free culture media and labeled with Vybrant^®^ DiD (Cell-Labeling Solution, V22887, Molecular Probes) following manufacturer protocol. Briefly, the cell suspension supplemented with DiD was incubated for 20 min at 37 °C. Following centrifugation, the supernatant was removed, and the stained cell pellets were washed in serum-free culture media. The cells were suspended in 50 µL of serum-free media and added to the material ink. The bioink was mixed with a sterile spatula before loading the syringe for printing. Cell printing was carried out using a 410 µm nozzle (Fisnar Europe, UK) fabricating 10 mm × 10 mm scaffolds with an alternating layer pattern (0°/90°). After the deposition, 3D-printed scaffolds were incubated for 10 min in sterile 100 mmol/L CaCl_2_ solution and then incubated at 37 °C and 5% CO_2_ balanced air. The cell-laden scaffolds for viability and functionality studies were printed in triplicates at each time point using DiD-stained and unstained bioinks, respectively.

#### Viability and functionality analysis

Cell viability was investigated after 1, 7, and 21 days of culture using confocal imaging, as previously described [8]. Briefly, the samples were washed twice with 1× HBSS. Scaffolds were then incubated in a diluted serum-free culture media solution of Calcein AM (C3099, Invitrogen, Thermo Fisher Scientific, UK) at 37 °C in 5% CO_2_ balanced air for 1 h, following the manufacturer’s protocol. Living cells were stained by both Calcein AM (green) and DiD (red). Non-metabolically active or dead cells were stained red by DiD. The scaffolds were imaged using a confocal scanning microscope (Leica TCS SP5, Leica Microsystems, Wetzlar, Germany), and the images were analyzed using ImageJ. Cell density was calculated by normalizing the number of viable cells with the volume of interest.

Cell-laden scaffolds were cultured in basal (α-MEM supplemented with 10% FBS and 1% Pen/Strep) and osteogenic (α-MEM supplemented with 10% FBS and 1% Pen/Strep, 100 µmol/L ascorbate-2-phosphate (AA2P, Sigma-Aldrich), 10 nmol/L dexamethasone (Dex, Sigma-Aldrich) and 10 nmol/L vitamin D (1α,25(OH)_2_D_3_, Sigma-Aldrich)) conditioned media.

Alkaline phosphatase (ALP) staining was carried out after 1, 7, and 21 days of culture at 37 °C in 5% CO_2_ balanced air. The samples were washed twice with 1× HBSS and fixed in 95% ethanol for 10 min. Scaffolds were left to dry while ALP staining solution, containing Naphthol (AS-MX Phosphate Alkaline Solution, 85–5, Sigma-Aldrich, UK) and Fast Violet Salt (F1631, Sigma-Aldrich, UK) solubilized in DW. Samples were incubated at 37 °C for 1 h with the ALP staining solution and the reaction was stopped by dilution of ALP solution with HBSS. The stained scaffolds were stored at 4 °C overnight and imaged the following day using a Zeiss Axiovert 200 (Carl Zeiss, Germany). Due to the limitations in molecular analysis for ALP activity, previously shown to interact with nanoclay disks [11], the ALP relative intensity and area percentage were quantified using ImageJ color inspector 3D and deconvolution to determine ALP intensity and levels.

#### Modeling absorption and release

Protein absorption and release study was carried out as previously reported [9]. Model proteins lysozyme from chicken egg (Sigma-Aldrich, UK) and bovine serum albumin (BSA, Sigma-Aldrich, UK) were solubilized in HBSS (Thermo-Fisher, UK) at 10 µg/mL and 100 µg/mL, respectively. To investigate the effect of the nanoclay particles on drug release, 3D scaffolds were printed using nanocomposite (LAB) and Laponite-free controls (alginate-bone-ECM (AB)) to allow the absorption of the compounds of interest after ionic crosslinking. The 3D-printed constructs (*n*=3) were soaked in lysozyme or BSA for 1 h, and their release was monitored over 24 h. BSA and lysozyme were quantified with a RAPID kit (Sigma-Aldrich, UK) using a GloMax Discover microplate reader (Promega). The supernatant was collected after 1, 2, 4, 8, 10, 20, and 24 h following adsorption. Collagenase D (from *Clostridium histolyticum*, Roche Diagnostics GmbH) was added 24 h after adsorption to stimulate material degradation and cargo release. BSA and lysozyme release was quantified after 1, 2, 4, 8, 10, 20, and 24 h.

### Chick chorioallantoic membrane (CAM) model

#### Scaffold fabrication for ex vivo vascularization

Scaffolds with nanoclay and LAP-free were 3D printed, crosslinked following 10 min exposure to 100 mmol/L CaCl_2_ and allowed to adsorb for 30 min with recombinant human vascular endothelial growth factor (rhVEGF 165, PeproTech, USA) at 100 μg/mL at 4 °C. 3D printed constructs were washed three times with 1× HBSS prior storage overnight at 4 °C.

#### Scaffold fabrication for ex vivo cell delivery and mineralization

Nanoclay-based and LAP-free 3D scaffolds were fabricated and implanted immediately after adsorption of recombinant human bone morphogenetic protein-2 (rhBMP-2) at 10 µg/mL for 30 min at 4 °C. Scaffolds were washed in 1× HBSS for three times before implantation.

#### CAM implantation, extraction, and Chalkley score

The CAM ex vivo model was used to evaluate vascularization and mineralization. Animal studies were conducted in accordance with Animals Act 1986 (UK), under Home Office Approval UK (PPL P3E01C456). Fertilized eggs were maintained in a rotating Hatchmaster incubator (Brinsea, UK) for 10 days at 37 °C and 60% humidity. 3D-printed scaffolds were implanted at day 10 post-fertilization. The implantation was carried out under a Class II laminar flow hood by creating a 2 cm^2^ window on the eggshells. The constructs were overlaid on the CAM, and the eggshell windows were sealed with sterile parafilm. The chicken eggs were incubated in a nonrotating incubator for seven days, and the developing chick embryos were inspected daily via candling to monitor their growth and viability. Following seven days of incubation, samples were harvested, and CAM integration was assessed using a stereomicroscope equipped with a digital camera (Canon Powershot G2). The overlap morphometry analysis was performed on the extracted samples as previously described [9]. Briefly, implanted samples were screened for vascular penetration by superimposing the Chalkley graticule and the afferent integrated CAM vasculature. The numbers of counted vessels colliding with the points on the graticule were assessed blinded to the study groups, and each sample counted three times. Samples were collected and fixed in 4% paraformaldehyde (PFA) overnight, before further processing for histological analysis. Afferent vessels diameter was evaluated following processing of stereomicroscope Images using ImageJ software analysis.

#### Mineral deposit formation

The deposition of mineral tissue was assessed using microcomputed tomography (micro-CT, Bruker Skyscan 1176). The samples fixed with 4% PFA were washed with HBSS before scanning and imaging using a pixel size of 35 µm, 65 kV, 385 µA, 0.7° rotation step, 135 ms exposure, and an aluminum (Al) filter of 1 mm. CT reconstructions were obtained via NRecon (Bruker) and quantitative analysis was performed using CTAn software (Bruker) to assess the average mineral density. Bone phantoms with predetermined bone density (0.25 g/cm^3^ and 0.75 g/cm^3^) were used as reference for calibrating the CT scans.

#### Histological analysis

Samples explanted from the ex vivo CAM assay were fixed in 4% PFA overnight at 4 °C, paraffin-embedded, and sliced using a microtome to produce 8-μm-thick sections. Goldner’s Trichrome, Alcian Blue & Sirius Red, and von Kossa staining was performed based on previous protocols [27]. Slides were imaged the following day using a Zeiss Axiovert 200 microscope (Carl Zeiss, Germany).

#### Statistical analysis

Experimental studies were evaluated by one-way and two-way analysis of variance (ANOVA) using Bonferroni’s multiple comparison tests. The analysis was performed using GraphPad Prism 9.0, and significance was set at ^*^p<0.05.

## Results

### Physicochemical and mechanical properties of nanoclay-based hydrogels

**Fig. 2:**
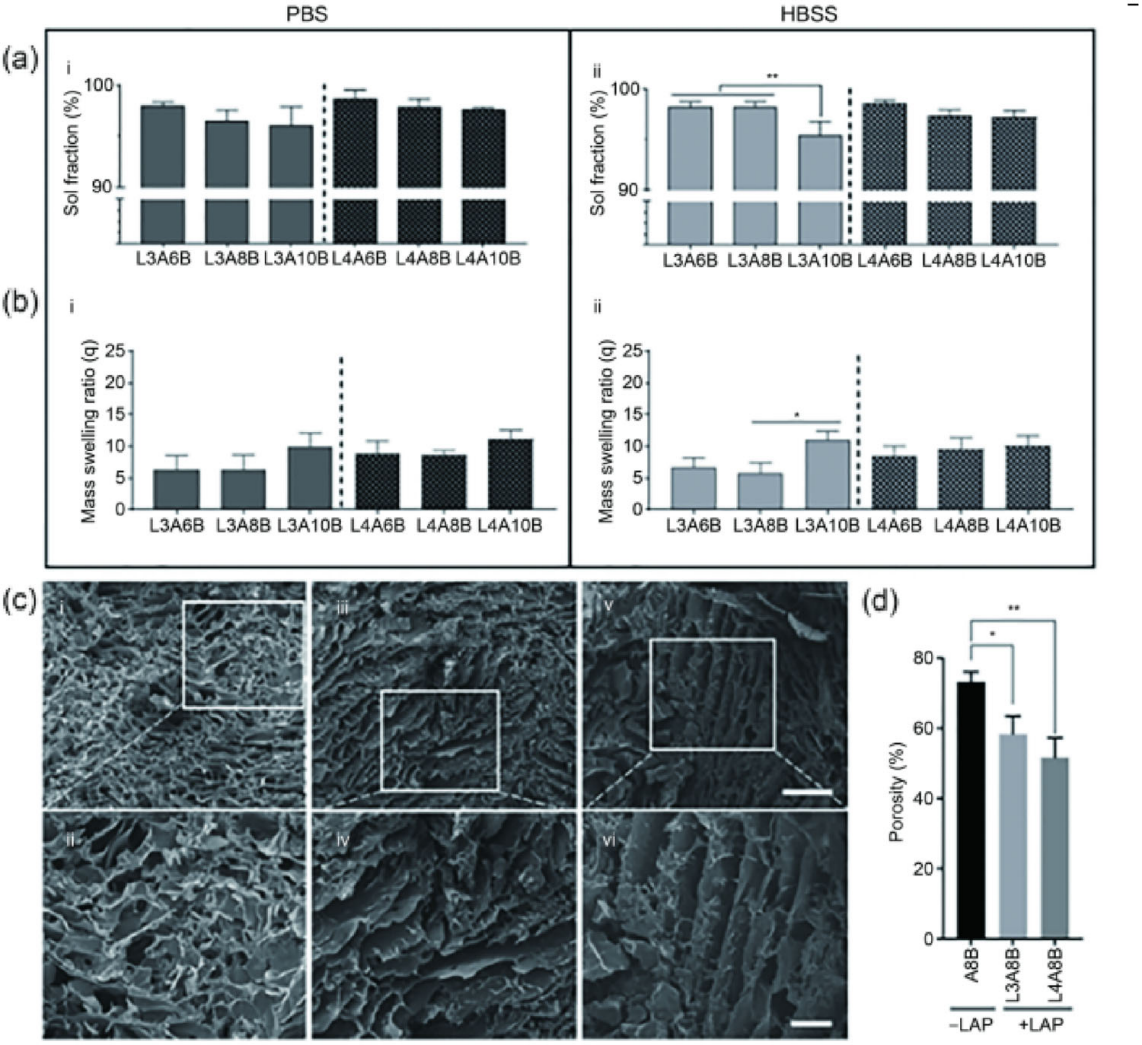
Physical characterization of composite inks. **a** Sol fraction and **b** mass swelling ratio analysis of scaffolds both in (a–i, b–i) PBS and (a–ii, b–ii) HBSS. **c** SEM micrographs of A8B (c–i, c–ii), L3A8B (c–iii, c–iv), and L4A8B (c–v, c–vi) scaffolds. (d) Porosity analysis of A8B, L3A8B, and L4A8B via ImageJ measurements. Scale bars: (c–i, c–iii) 500 µm, (c–ii, c–iv) 200 µm. Statistical significance was assessed by unpaired *t* test (Welch-corrected). Data are presented as mean±standard deviation, *n*=3, ^*^p<0.05, ^**^p<0.01. PBS: phosphate-buffered saline; HBSS: Hank’s balanced salt solution; SEM: scanning electron microscopy

The physicochemical properties of bone-ECM nanocomposite inks were investigated following printing and maintenance in PBS and HBSS buffers. A range of material composites was explored by varying the LAP concentration from 3% to 4% (mass fraction), and the alginic acid inclusion between 6% and 10% (mass fraction). The concentration of bone-ECM was kept constant as the percentage of inclusion (10 mg/mL) was fixed. The sol fraction (Fig. 2a) decreased with the increase in alginate concentration in PBS (Fig. 2a–i) and HBSS (Fig. 2a–ii), with a significant reduction between L3A6B and L3A10B. This was consistent with results obtained for alginate controls both in sol fraction (Fig. S1a in Supplementary Information) and mass swelling ratio data generated (Fig. S1b in Supplementary Information).

The mass swelling ratio (*q*) revealed a non-significant increase in the swelling as the alginate fraction was increased in the nanocomposite ink in both PBS (Fig. 2b–i) and HBSS (Fig. 2b–ii). Controls in PBS (Fig. S1c in Supplementary Information) and HBSS (Fig. S1d in Supplementary Information) showed a significant decrease in sol fraction and a proportional increase in swelling ratio with an increase in LAP content. The microstructural arrangement of LAB was investigated via SEM imaging. The porosity of the LAP-free (Figs. 2c–i and 2c–ii) samples was significantly higher than the 3% LAP (Figs. 2c–iii and 2c–iv) and 4% LAP (Figs. 2c–v and 2c–vi) samples.

**Fig. 3:**
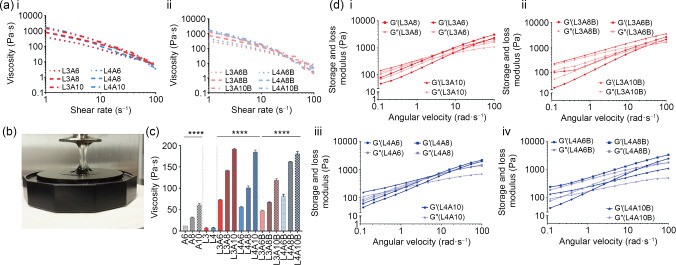
Rheological properties of the nanoclay-based bone-ECM inks. **a** Viscosity over shear rate study of a series of nanoclay-based materials (a–i) in absence or (a–ii) inclusion of bone-ECM. **b** LAB gel over rheometer plates showing viscoelastic behavior. (c) Viscosity comparison at a fixed shear rate (10 s^–1^). **d** Storage and loss moduli of nanoclay-based materials (d–i, d–iii) without and (d–ii, d–iv) when blended with bone-ECM. Statistical significance was assessed by one-way ANOVA. Data are presented as mean±standard deviation, *n*=3, ^∗∗∗∗^*p*<0.0001. ECM: extracellular matrix; LAB: Laponite-alginate-bone-ECM; ANOVA: analysis of variance

Rheological measurements of LAB inks were determined to investigate the printing capacity and stability following extrusion. Viscosity was measured as a function of shear rate (Fig. 3a). We found that the viscoelastic properties and nanoclay concentration were correlated as the viscosity was higher at different shear rates compared to controls (Fig. S2a in Supplementary Information). LAP inclusion augmented viscosity in all blends (Figs. 3a and 3b) across the range of shear rates examined. The increase in LAP concentration was found to significantly enhance viscous moduli of nanocomposites at a fixed shear rate (Fig. 3c), confirming the ability of the nanoclay to enhance the viscous properties of poorly viscous polymers. Storage and loss moduli of the nanocomposite blends (Fig. 3d, i–iv) displayed a viscoelastic behavior compared to the controls (Fig. S2b in Supplementary Information) and stabilized as the angular momentum was increased.

### Printing characterization of nanocomposite bone-ECM ink

**Fig. 4:**
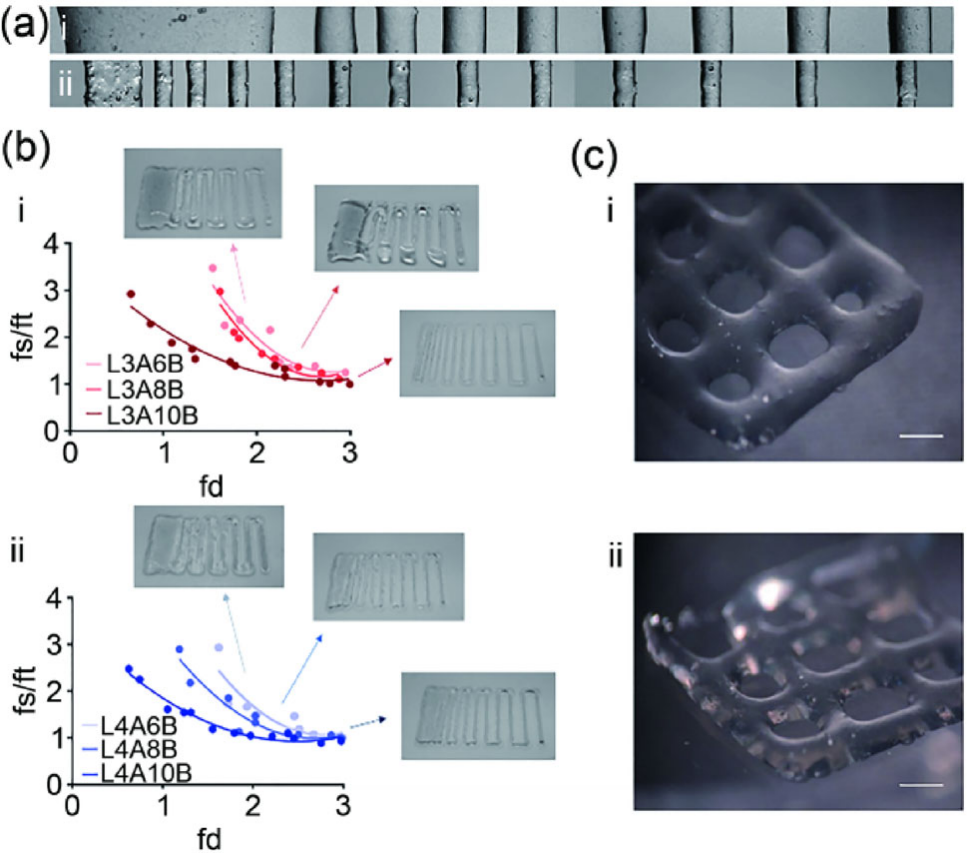
Printing fidelity of nanocomposite bone-ECM inks. **a** Filament fusion test was carried out with (a–i) AB and (a–ii) LAB inks. **b** Measurements of the filament fusion tests performed with (b–i) 3% and (b–ii) 4% LAP composite inks. **c** Micrographs of scaffolds printed with (c–i) 3% and (c–ii) 4% LAP-based inks. Scale bar: 1 mm. AB: alginate-bone-ECM; LAB: Laponite-alginate-bone-ECM; LAP: Laponite; fs: fused segment length; ft: filament thickness; fd: filament distance

To evaluate the printing resolution and shape fidelity of the nanocomposite bone-ECM inks, a regular pattern with increasingly spaced fiber distances was generated. A custom G code was written to investigate the ability of the inks of different LAP and alginate compositions to be deposited as fine fibers at increments of 200 µm. The length of the fused portion of printed fibers (fs) and fiber thickness (ft) were measured, and the resulting quotients were plotted against fiber distance (fd). Micrographs (Fig. 4) were analyzed following AB (Fig. 4a) and LAB (Fig. 4b) deposition.

The resulting analysis indicated that the inclusion of increasingly greater percentage of alginate (6%, 8%, and 10%) was included in inks at fixed Laponite concentrations of 3% (Fig. 4b–i) and 4% (Fig. 4b–ii), and the printability of the nanocomposite formulation was enhanced. Increases in fiber distances caused a rapid decrease in the measured values, confirming the enhanced shape fidelity and resolution. The nanocomposite bone-ECM ink comprising 3% nanoclay was found to be printable and could be consistently deposited till up to four layers (Fig. 4c–i). The inclusion of an increased percentage of nanoclay (4%) facilitated the printing of increasingly stable scaffolds (Fig. 4c–ii) at low alginate concentrations. Consequently, a concentration of 4% LAP and 8% alginate was used for the functional studies.

### Nanocomposite bone-ECM inks support HBMSC retention, viability, and functionality after printing

**Fig. 5:**
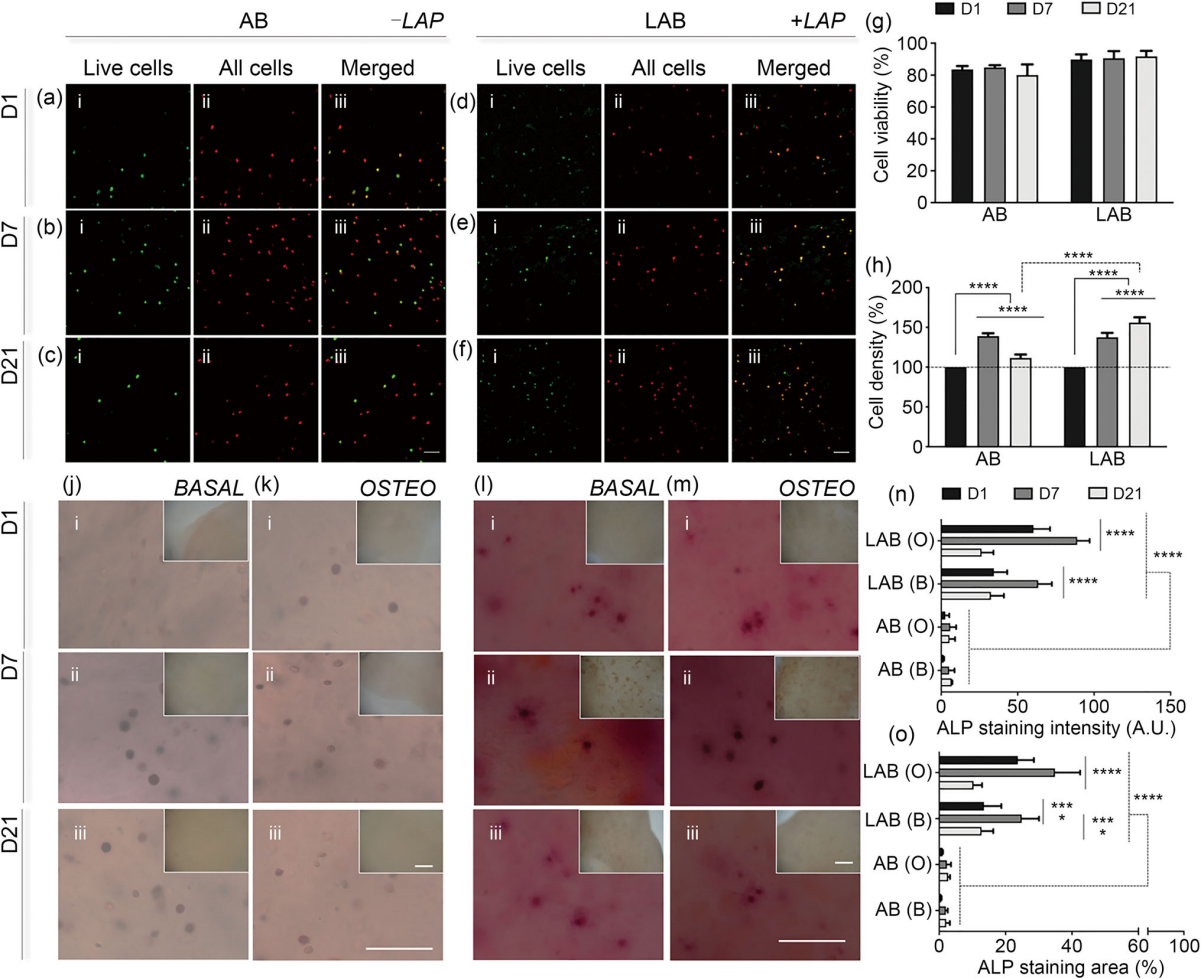
HBMSC viability and proliferation post-printing. Live/dead assay was performed on 3D-printed **a**–**c** AB and **d**–**f** LAB scaffolds at Days 1, 7, and 21. **g** Cell viability and **h** density quantification following ImageJ analysis. **j**–**m** ALP staining of 3D bioprinted scaffolds following cultivation in basal (AB, j; LAB, l) and osteogenic (AB, k; LAB, m) media conditioning complete with acellular control (insets). **n** ALP intensity and **o** area coverage percentage. Scale bars: **a**–**f** 100 µm, **j**–**m** 50 µm (samples), 250 µm (acellular controls). Statistical significance was determined using two-way ANOVA. Data are presented as mean±standard deviation, *n*=3, ^∗∗∗∗^*p*<0.0001. HBMSC: human bone marrow stromal cell; AB: alginate-bone-ECM; LAB: Laponite-alginate-bone-ECM; ALP: alkaline phosphatase; ANOVA: analysis of variance; O: osteogenic; B: basal

To evaluate their viability, the HBMSCs were encapsulated in nanoclay-free ink as control and printed in the nanocomposite bone-ECM hydrogel followed by 3D deposition. Viability was investigated in control (Figs. 5a–5c) and nanocomposite LAB ink (Figs. 5d–5f) using a live/dead assay. Cells remained viable in 3D printed scaffolds (Fig. 5g) at Day 1 ((83.50±2.23)% and (89.82±3.17)%), Day 7 ((84.78±1.46)% and (90.53±4.50)%), and Day 21 ((80.05±6.67)% and (91.72±3.48)%) in AB and LAB, respectively. The proliferation of printed HBMSCs was subsequently quantified over 21 days of culture in vitro. HBMSCs printed in LAP-free ink were observed to proliferate for up to 7 days post-printing comparable to nanocomposite ink samples. After 21 days, HBMSC density decreased significantly in AB ink, compared to LAB material, which was found to sustain a low but steady cell growth over 21 days.

To confirm the osteogenic potential of specific nanocomposite blends, ALP staining and analysis were performed on HBMSCs cultured on two-dimensional films of LAP-based bone-ECM hydrogels (Figs. S3–S5 in Supplementary Information) and on culture plastic.

Cell culture in basal and osteogenic media revealed an enhanced temporal ALP deposition by HBMSCs on LAB blends with varying Laponite concentrations (L3 (3% Laponite) and L4 (4% Laponite) in Figs. S3 and S4, respectively, in Supplementary Information) over 7 days compared to controls (Fig. S5 in Supplementary Information). LAP materials could support HBMSC differentiation at early stages (Day 1) when seeded at high density.

HBMSC-laden bone-ECM inks were 3D-printed and cultured for up to 21 days in basal and osteogenic culture media. Printed nanoclay-free bioink (AB), compared to cell-free controls and cell-laden LAB scaffolds, showed limited expression of ALP both at Days 1 (Fig. 5j–l, m–i), 7 (Fig. 5j–ii, m–ii), and 21 (Figs. 5j–iii, m–iii) in basal and osteogenic conditions. The inclusion of LAP within the material ink was found to elicit a significantly (*p*<0.0001) enhanced intensity (Fig. 5n) and ALP area deposition (Fig. 5o) up to 21 days, in both basal and osteogenic media. We note that the diffuse staining in the LAB gels is likely to be due to clay uptake of the ALP dye product originating from the embedded cells which are, themselves strongly and specifically stained. In the absence of cells, no equivalent staining is observed.

### The inclusion of nanoclay in bone-ECM inks improved drug retention and sustained release

To evaluate the ability of nanoclay bone-ECM inks to retain biologics/compounds of interest, such as lysozyme, BSA, BMP-2, and VEGF, the agents were adsorbed onto 3D-printed scaffolds for 24 h. Following adsorption, in vivo conditioning was simulated by adding a collagenase solution to trigger material degradation to enable the release of the absorbed cargo.

The ability of LAB and LAP-free (AB) scaffolds to absorb and retain biologics of interest was examined by quantifying the kinetic release of lysozyme (Fig. S6a in Supplementary Information) and BSA (Fig. S6b in Supplementary Information) over 48 h. LAB adsorbed a greater concentration of both lysozyme and BSA. Collagenase inclusion after 24 h of adsorption triggered the release of the cargo agents, enabling LAP-based scaffolds to retain a significantly larger proportion of lysozyme and BSA compared to AB for up to 24 h.

**Fig. 6:**
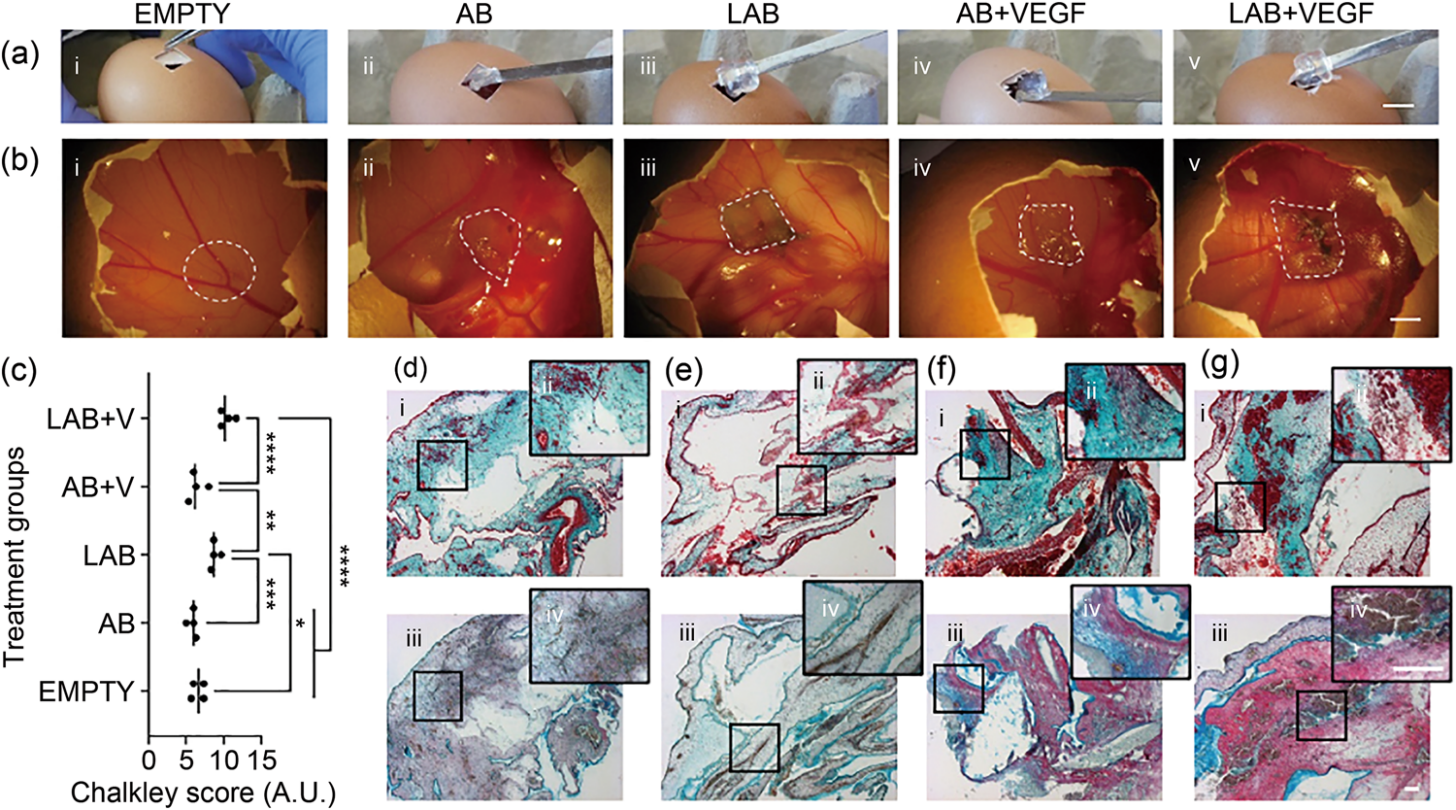
Nanoclay-based inks support sustained release of VEGF in the CAM model. Macrographs during **a** sample implantation and **b** retrieval: (i) empty, (ii) AB, (iii) LAB, and VEGF-loaded (iv) AB and (v) LAB 3D-printed scaffolds. **c** Chalkley score of vascularized samples and controls. **d**–**g** Histological micrographs of samples stained for (i, ii) Goldner’s Trichrome and (iii, iv) Alcian Blue & Sirius Red. Statistical significance was assessed using one-way ANOVA. Data are presented as mean±standard deviation, *n*=4, ^*^p<0.05, ^**^p<0.01, ^***^p<0.001, ^****^p<0.0001. Scale bars: **a**, **b** 10 mm, **d**–**g** 100 µm. VEGF: vascular endothelial growth factor; CAM: chick chorioallantoic membrane; AB: alginate-bone-ECM; LAB: Laponite-alginate-bone-ECM; ANOVA: analysis of variance

To investigate the ability of the 3D-printed LAB scaffold to retain and localize growth factors of interest for bone regeneration, VEGF was adsorbed by 3D-printed LAB and AB controls and implanted in the developing chick embryo CAM (Fig. 6a). The explanted groups were observed to be highly vascularized (Fig. 6b), evidenced by Chalkley score analysis (Fig. 6c). The number of blood vessels on the VEGF-laden LAB scaffolds was significantly higher (*p*<0.0001) than those on the scaffolds implanted with empty, AB-VEGF, and VEGF-free (AB and LAB) controls. Histological analysis (Figs. 6d–6g) confirmed the potential of VEGF-loaded samples to promote blood vessel formation as well as a higher deposition of collagenous matrix in LAP-based VEGF-loaded groups.

**Fig. 7:**
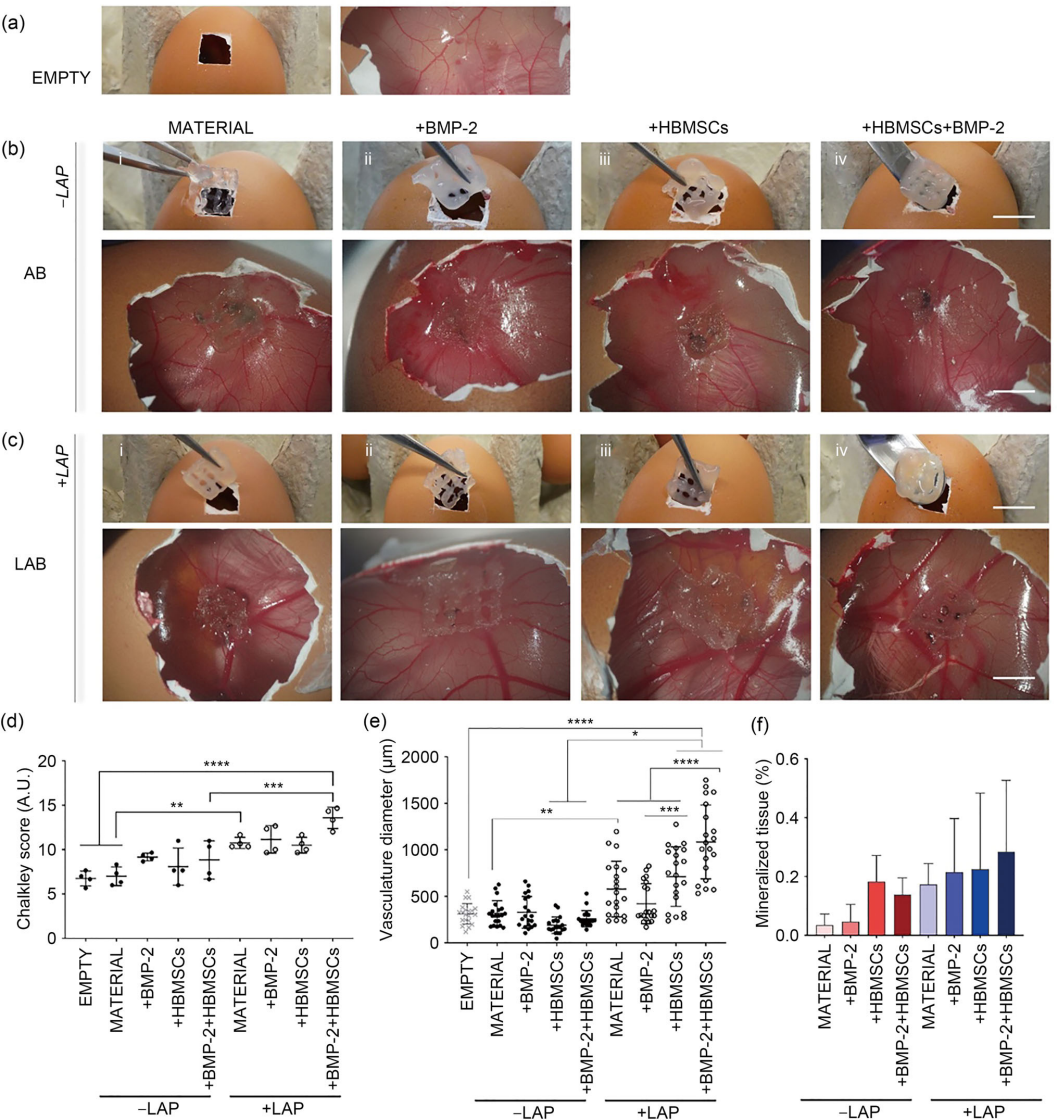
Nanocomposite bone-ECM scaffolds support mineralization ex vivo. **a** Macro- and micro-graphs of empty control. Implanted and explanted **b** LAP-free and **c** LAP-loaded 3D (i) material (drug- and cell-free) control, (ii) BMP-2 loaded, (iii) cell-loaded, and (iv) BMP-2 and cell loaded scaffolds. **d** Chalkley score of implanted samples and control after 7 d of culture. **e** Quantitative analysis of afferent vascular supply to implanted scaffolds before extraction. **f** Micro-CT analysis of implanted scaffolds following 7 d of incubation in a CAM model. Scale bars: **a**–**c** 10 mm. Statistical significance was assessed by one-way ANOVA. Data are presented as mean±standard deviation, *n*=4, ^*^p<0.05, ^**^p<0.01, ^***^p<0.001, ^****^p<0.0001. LAP: Laponite; 3D: three-dimensional; BMP-2: bone morphogenetic protein-2; micro-CT: microcomputed tomography; CAM: chick chorioallantoic membrane; ANOVA: analysis of variance; HBMSCs: human bone marrow stromal cells

Additional CAM analysis was undertaken to explore the synergistic effect of HBMSCs and BMP-2 in an ex vivo scenario. Compared to empty controls (Fig. 7a), the implanted 3D-printed LAP-free (Fig. 7b) and nanoclay-based (Fig. 7c) constructs were observed to be fully integrated.

Blood vessels were quantified using the Chalkley score method (Fig. 7d). HBMSC-laden LAB scaffolds containing BMP-2 were highly vascularized with more blood vessels than HBMSC-laden BMP-2-loaded AB scaffolds (*p*<0.001), empty controls, and LAP-free acellular and BMP-2-free scaffolds (*p*<0.0001). LAB scaffolds were found to promote significant vascularization compared to AB scaffolds (*p*<0.01).

**Fig. 8:**
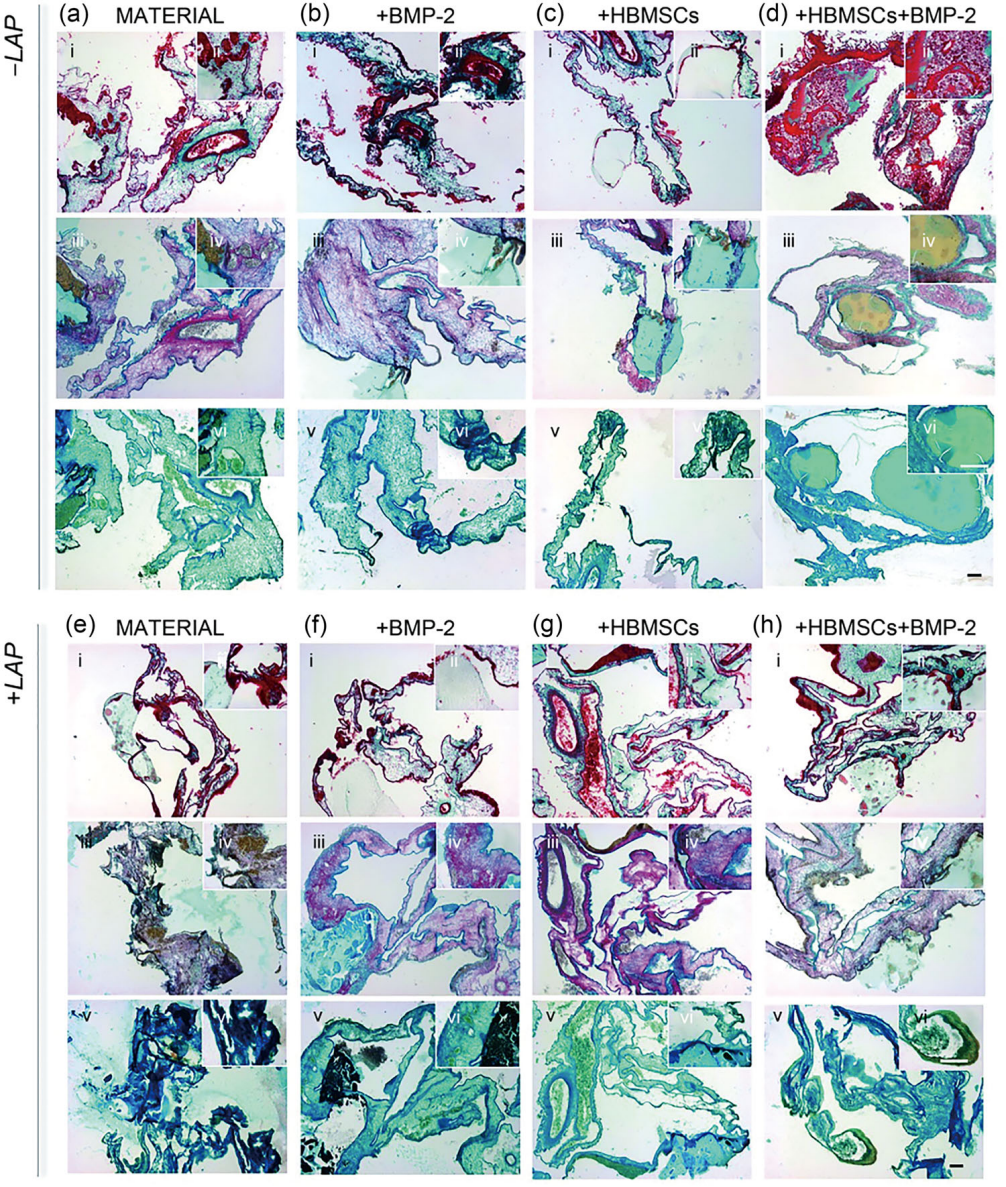
CAM implantation of 3D-printed scaffolds containing BMP-2 and HBMSCs. **a**–**d** LAP-free and **e**–**h** LAP-loaded groups are stained for (i, ii) Goldner’s Trichrome, (iii, iv) Alcian Blue & Sirius Red, and (v, vi) von Kossa. Scale bars: 100 µm. CAM: chick chorioallantoic membrane; 3D: three-dimensional; BMP-2: bone morphogenetic protein-2; HBMSCs: human bone marrow stromal cells; LAP: Laponite

The vessel diameters were measured *in ovo* before isolation (Fig. 7e). The acellular and biologic LAB scaffolds were observed to be significantly larger (*p*<0.01) than 3D-printed AB materials. The inclusion of LAP nanosilicate disks significantly enhanced blood vessel diameter (*p*<0.0001) when combined with BMP-2, HBMSCs, and both. Thus, the synergistic combination of HBMSCs and BMP-2 was found to stimulate the formation of larger vessels (1 mm) compared to AB and LAB control scaffolds (*p*<0.0001). Micro-CT analysis of explanted 3D scaffolds (Fig. 7f) revealed the presence of mineralized tissue although this was not significantly greater than the controls (acellular and BMP-2-free printed inks). Histological analysis (Fig. 8) revealed vascularization in the LAP-free (Figs. 8a–8d) and LAP-based constructs (Figs. 8e–8h). Implanted nanoclay-free 3D constructs loaded with BMP-2 and HBMSCs (Figs. 8d–i and 8d–ii) resulted in leakage of vessels in the chorioallantoic membrane, resulting in extensive penetration of vessels accompanied by erythrocytes dispersion across the implant. A collagenous matrix was present in cell-laden groups (both LAP-free and LAP-based), demonstrating the functionality of HBMSCs after seven days of implantation. LAP-based controls stained positive for the mineral stain von Kossa compared to LAP-free controls.

## Discussion

A variety of manufacturing strategies, including electrospinning [28] as well as implantation approaches, such as non-invasive injection [29], have been recently exploited for bone repair. However, biofabrication technologies have rapidly advanced the engineering of 3D substitutes for the repair of damaged and diseased skeletal tissue, through the generation of new complex 3D architectures. However, the lack of functional inks, capable of supporting cell growth and differentiation post-printing and, ultimately, to regenerate skeletal defects, remains an unresolved challenge. The current study details the incorporation of human demineralized and decellularized bone-ECM in combination with nanosilicate (Laponite^®^) particles and alginate polymer for the design of a bioactive ink. The addition of both LAP and alginate to a human bone decellularized and demineralized ECM was found to stabilize the sol fraction and mass swelling ratio at low polymeric content.

The engineering of nanocomposite materials, incorporating functional fillers capable of modifying physical properties (e.g., thixotropic behavior), compound interactions (e.g., drug localization), and biological functionality (e.g., cell spreading), has supported the fabrication of cell-laden constructs for the active repair of skeletal defects. Nevertheless, the sole inclusion of nano-fillers does not guarantee the engineering of a functional microenvironment for stem/progenitor cell proliferation and differentiation [30]. Decellularized ECM provides a particularly attractive approach to mimic the native tissue-specific microenvironment. Recently, several studies [17, 31, 32] have demonstrated the ability to print non-human decellularized ECM (particularly cardiac [17, 31] and hepatic [32] tissues) in combination with clay nanodisks, demonstrating the beneficial inclusion of nanoclay fillers to drastically improve printability and printing fidelity. Nevertheless, the animal-sourced decellularized materials (mainly porcine), while providing a similar collagen, glycosaminoglycans, and growth factors content, can still generate an immune response. Thus, human-based decellularized tissue has come to the fore as an ideal biomaterial for tissue regeneration. In particular, ECM components after digesting demineralized and decellularized human cancellous bones offer significant potential to improve cellular responses. We have further characterized the physicochemical and biochemical properties of the human decellularized bone-ECM [14].

The investigation of the microstructure of the LAB material revealed a difference in porosity. LAP-based inks were found to be less porous as the positive rim charge of the nanoparticles can closely interact with negatively charged alginate and collagen-abundant bone-ECM components.

This was further confirmed by rheological studies, demonstrating a significant increase in viscous properties with the inclusion of nanoclay particles within the composites behavior already observed in a number of previous studies [9, 11, 30]. Indeed, LAP nanoparticles hold the ability to closely interact electrostatically with polymeric chains closely, reducing the distance between the biomaterial networks, thus increasing viscosity and ultimate mechanical properties. The ability of LAP to promote mineralization together with the retention and localization of biological agents has been previously demonstrated [33], making nanosilicate materials an attractive biomaterial for bone tissue regeneration. Moreover, the shear-thinning properties of LAP-based inks have been found essential for 3D bioprinting applications of skeletal implants [11]. The control over viscoelastic properties and the influence on printability were demonstrated by the filament fusion test. The results highlighted that increased LAP concentration can significantly influence the printability over several stacked layers. However, alternate 0°/90° patterning was observed to be influenced by the post-printing relaxation of the viscous properties, with an increase in shape fidelity directly correlated with increase in LAP content, in agreement with a previous report [8, 34].

The overall viscoelastic properties of the LAB ink were tuned to allow HBMSC printing. LAP-based cell-laden scaffolds supported HBMSC proliferation over 21 days compared to LAP-free control as previously reported [9, 11]. The cell retention ability of LAB scaffolds was a likely result of the enhanced viscoelastic properties compared to AB constructs, preserving the integrity of the overall printed construct over time, and avoiding the release of cell material from the degrading fibers. Furthermore, in agreement with previous results [8, 10], LAP inclusion was found to aid HBMSC differentiation toward bone lineage, as highlighted by the ALP staining micrographs. The 3D printing of HBMSCs reduced spatial spreading of encapsulated stromal cells and facilitated a functional response with intense ALP expression in vitro as well as collagen deposition following ex vivo implantation, as previously reported [9, 10].

The addition of LAP nanodisks facilitated the local release of ALP over 21 days. As previously reported [10, 11], nanocomposite inks stimulated ALP deposition immediately post-printing (Day 1), supporting the rapid formation of skeletal-specific biomimetic scaffolds. Thus, the nanosilicate inclusion detailed in these studies is ideal for in vitro bone modeling in combination with alginate, specifically supporting the 3D deposition, while the addition of bone-ECM enhanced the functionality of the printed scaffold. ALP was found to be expressed ubiquitously in nanoclay-based sample groups as previously reported [9–11]. The deposition and intensity of ALP were correlated with the concentration of LAP in the composite and were dependent on the concentration and presence of nanoclay over 21 days. The ALP staining in LAP-only and LAP-bone-ECM samples was present from Day 1 to Day 7 both in basal and osteogenic conditions. Nevertheless, the presence of alginate appeared to alter the morphology of seeded HBMSCs as previously reported [8, 30] as the HBMSCs developed a rounded morphology with concomitant expression of ALP from the first day of culture.

We investigated the ability of nanoclay-modified bone-ECM scaffolds to localize biological agents of interest within a preclinical scenario using the CAM assay. Ex vivo implantation of 3D-printed LAP-based bone-ECM constructs demonstrated that these new biomimetic materials can be blended to support angiogenesis, with vessels forming within seven days of implantation due to the localization of GFs within the matrix. In the absence of nanoclay, no significant response in vessel ingrowth was observed, even in the presence of VEGF. As previously reported [11] and demonstrated here by the controlled release of BSA and lysozyme, the absence of nanoclay and lack of adsorptive potential of the scaffolds typically result in the burst release of encapsulated factors and pharmaceutical agents. Notably, the inclusion of nanoclay which can bind and enhance the activity of growth factors, did elicit an angiogenic response even without any exogenous VEGF. The retention of VEGF was found to stimulate vessel ingrowth in LAP-based implants, as previously demonstrated for nanoclay-based constructs [9]. Furthermore, this study illustrated the synergistic interaction of a nanocomposite (LAP, human bone-ECM, and alginate) ink microenvironment for the proliferation and functionality of HBMSCs. Indeed, the deposition of cell-laden BMP-2-loaded constructs enhanced mineralization and vascularization. In addition, the diameter of the CAM blood vessels was significantly increased when LAP was combined with alginate and bone-ECM. Although this phenomenon is well documented for local BMP-2 exposure [11], it is less clear in drug-free implants. Thus, bone-ECM combined with LAP was found to support angiogenesis, providing a platform to stimulate the vascularization of a skeletal TE construct. Angiogenesis is fundamental to osteogenesis and the osteogenic response in fracture repair. This has been evidenced using VEGF165, a potent angiogenic factor that mediates osteogenesis and bone repair and modulates angiogenesis, chondrocyte apoptosis, cartilage remodeling, osteoblast function, and endochondral growth plate ossification in endochondral bone formation [35, 36]. VEGF and BMP-2 can synergistically stimulate neovascularization and bone growth. Our ongoing work aims to explore the underlying biochemical mechanisms. In vivo studies in mice were considered but the data from the CAM model provided compelling information about the novel ECM-based scaffold material. We felt at this time that in vivo studies in mice would only provide minimal further validation, hence trying to minimize the use of animal studies in accordance with the 3Rs (reduce, refine, and replace) that ex vivo investigation was carried out. We are cogniscent that the use of human bone-ECM tissue could be initially limited by immunological issues impacting clinical translation. Nevertheless, the possibility of generating a patient-specific decellularized bone ink, harnessing the patients’ own skeletal tissue, offers an exciting opportunity for a personalized medicine approach to aid bone repair using a human bone-ECM biomimetic engineered tissue substitute.

## Conclusions

The design of biomimetic functional biomaterials for skeletal tissue engineering is a key goal in aiding bone repair. Xenogeneic ECM matrices containing GFs and native polymers can be applied to effectively repair damaged skeletal tissue. However, issues around immunogenicity, synthesis, and limited mechanical properties have limited the use of ECM matrices for 3D bioprinting purposes.

This study sought to harness human bone-ECM in combination with alginate and nanoclay particles to fabricate implantable constructs capable of supporting and promoting bone repair. Our results show that LAP limited the swelling of printable inks, enabled tuning of rheological properties, and allowed the printing of self-sustained 3D structures comprising bone-ECM with an ultra-low polymeric concentration. This novel human bone-ECM ink supported the deposition of HBMSCs, maintaining their viability and supporting the proliferation and differentiation along the osteogenic lineage in vitro and ex vivo. LAP-based scaffolds were found to retain VEGF or BMP-2 in an ex vivo CAM model, highlighting the ability to sustain angiogenic and osteogenic development, which is important in endochondral ossification and skeletal repair. Future studies, outside the scope of the current work, will examine the in vivo application of the ECM-based 3D bioprinted skeletal construct, targeting the functional repair of fracture and calvarial preclinical models of bone repair. Additional improvements are in development to strengthen the overall 3D-printed structure that is currently non-supportive of skeletal regeneration within load-bearing defects.

In summary, this study demonstrates the 3D patterning of a novel nanocomposite ink containing human bone-ECM components, capable of supporting HBMSC viability and sustaining growth factor release with potential application in bone repair.

### Supplementary Information

Below is the link to the electronic supplementary material.

Supplementary file1 (DOCX 1676 kb)
