# Supplementary material for: Biofabrication of nanocomposite-based scaffolds containing human bone extracellular matrix for the differentiation of skeletal stem and progenitor cells
Source: Biodes Manuf. 2024 Mar 5;7(2):121–36. doi: 10.1007/s42242-023-00265-z (PMC10937808; doi:10.1007/s42242-023-00265-z)
Supplement: Supplementary file 1 — Supplementary file1 (DOCX 1676 kb) [file 42242_2023_265_MOESM1_ESM.docx]

# Supplementary Information


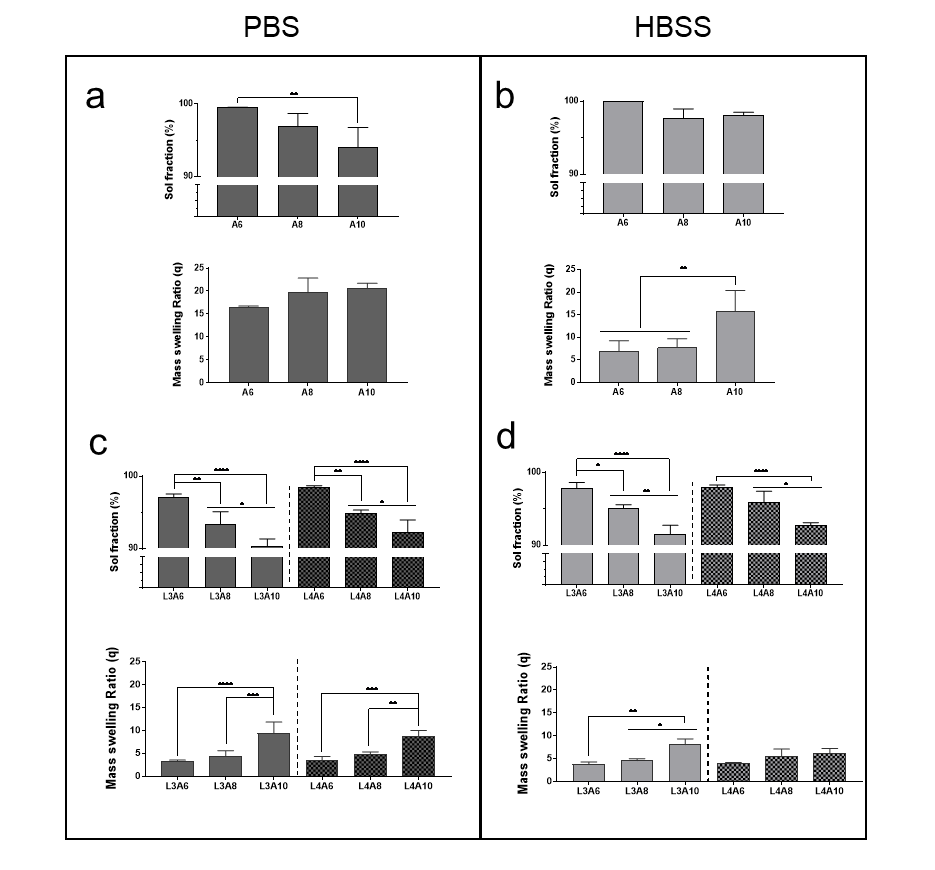


**Supplementary Figure 1.** Physical investigation of control composite inks. Sol fraction and mass swelling ratios of alginate and nanoclay-modified alginate controls in PBS (a,c) and HBSS (b,d), respectively. Statistical significance was assessed by one-way ANOVA. Mean ± S.D. n=3, ∗p<0.05, ∗∗p<0.01, ∗∗∗p<0.001, ∗∗∗∗p<0.0001


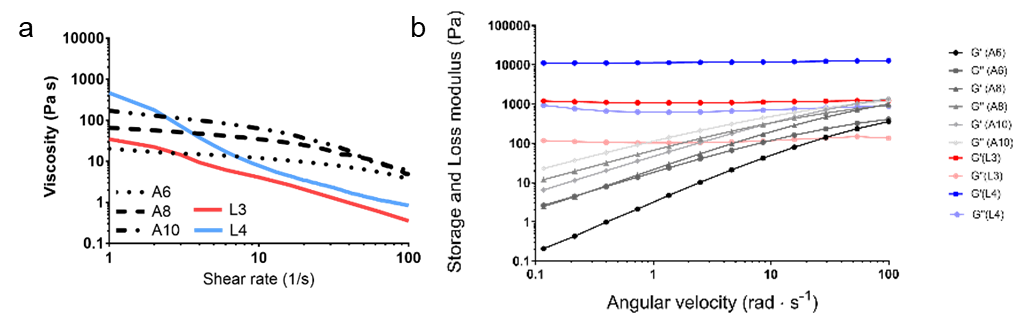


**Supplementary Figure 2**. Rheological characterization of control materials. Viscosity (a) and storage and loss moduli (b) of the alginate and Laponite controls.


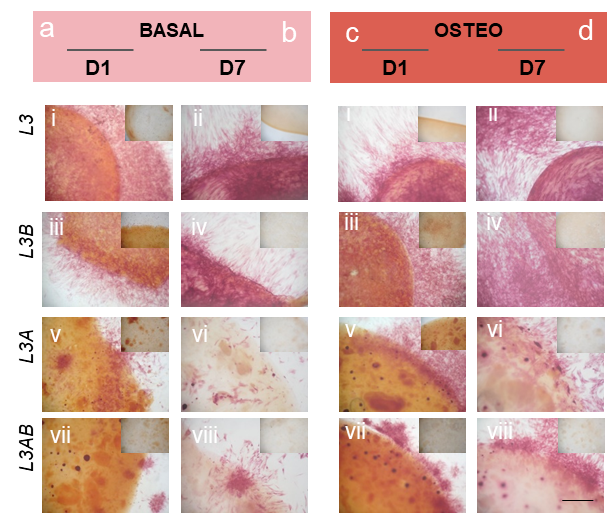


**Supplementary Figure 3.** ALP staining of HBMSCs seeded on 3% w/v nanoclay (Laponite) and composites (Laponite-bone-ECM (L3B), Laponite-alginate (L3A), Laponite-alginate-bone-ECM (L3AB)) at day 1 and 7 both cultured in basal (a,b) and osteogenic (c,d), respectively. Scale bars: 250 µm


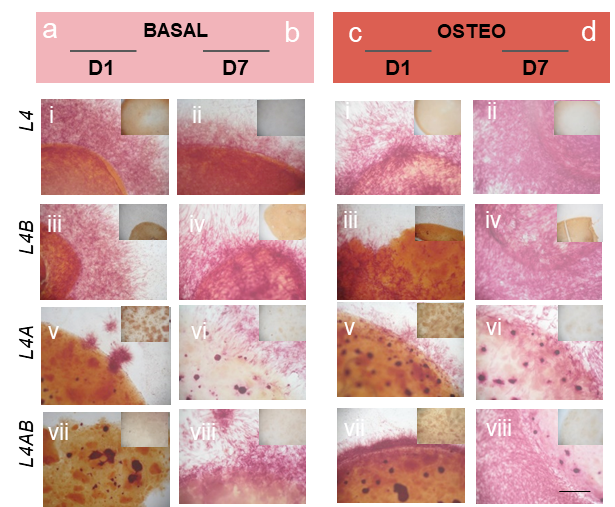


**Supplementary Figure 4**. ALP staining of HBMSCs seeded on 4% w/v nanoclay (Laponite) and composites (Laponite-bone-ECM (L4B), Laponite-alginate (L4A), Laponite-alginate-bone-ECM (L4AB)) at day 1 and 7 cultured in basal (a,b) and osteogenic (c,d) media, respectively. Scale bars: 250 µm


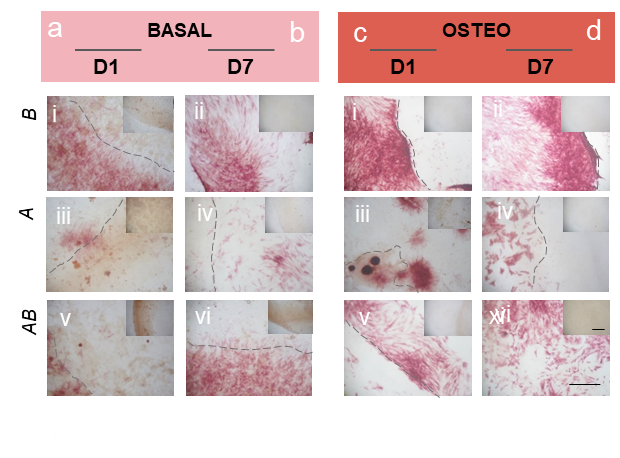


**Supplementary Figure 5**. ALP staining of HBMSCs seeded on bone ECM (B), alginate (A) and composite (AB) at day 1 and 7 cultured in basal (a,b) and osteogenic (c,d) media, respectively. Scale bars: 250 µm


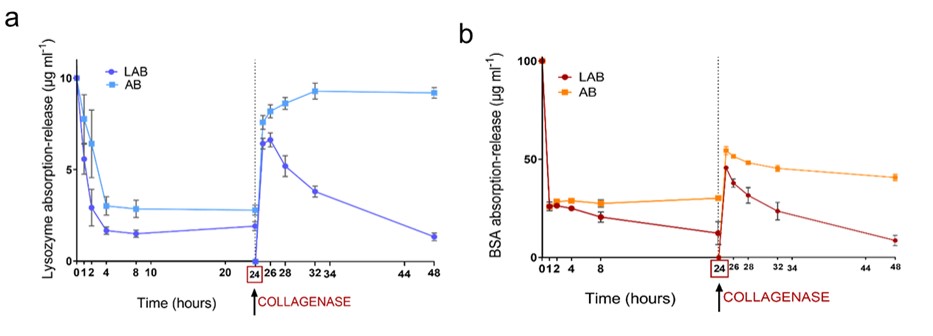


**Supplementary Figure 6.** Absorption/release of model proteins, lysozyme (a), and bovine serum albumin (b) using collagenase-mediated release following 24-hour absorption.
